# The Biggest Challenges Resulting from the COVID-19 Pandemic on Gender-Related Work from Home in Biomedical Fields—World-Wide Qualitative Survey Analysis

**DOI:** 10.3390/ijerph19053109

**Published:** 2022-03-06

**Authors:** Eva Bezak, Kristin V. Carson-Chahhoud, Loredana G. Marcu, Magdalena Stoeva, Lenka Lhotska, Gilda A. Barabino, Fatimah Ibrahim, Eleni Kaldoudi, Sierin Lim, Ana Maria Marques da Silva, Peck Ha Tan, Virginia Tsapaki, Monique Frize

**Affiliations:** 1 Cancer Research Institute, University of South Australia, Adelaide, SA 5001, Australia; eva.bezak@unisa.edu.au (E.B.); kristin.carson-chahhoud@unisa.edu.au (K.V.C.-C.); 2 Australian Centre for Precision Health, University of South Australia, Adelaide, SA 5001, Australia; 3 School of Medicine, University of Adelaide, Adelaide, SA 5001, Australia; 4 Faculty of Informatics and Science, University of Oradea, 1 Universitatii Str., 410087 Oradea, Romania; 5 Department of Diagnostic Imaging, Medical University of Plovdiv, 4002 Plovdiv, Bulgaria; ms_stoeva@yahoo.com; 6 Faculty of Biomedical Engineering, Czech Technical University in Prague, 160 00 Prague 6, Czech Republic; lenka.lhotska@cvut.cz; 7 Olin College of Engineering, Needham, MA 02492, USA; presidentbarabino@olin.edu; 8 Department of Biomedical Engineering, Centre for Innovation in Medical Engineering, Faculty of Engineering, Universiti Malaya, Kuala Lumpur 50603, Malaysia; fatimah@um.edu.my; 9 School of Medicine, Democritus University of Thrace, 69100 Alexandroupoli, Greece; kaldoudi@med.duth.gr; 10 School of Chemical and Biomedical Engineering, Nanyang Technological University, Singapore 637457, Singapore; slim@ntu.edu.sg; 11 School of Technology, Pontifical Catholic University of Rio Grande do Sul, PUCRS, Porto Alegre 90619-900, Brazil; ana.marques@pucrs.br; 12 School of Engineering, Ngee Ann Polytechnic, Singapore 599489, Singapore; tan_peck_ha@np.edu.sg; 13 Medical Physics Department, Konstantopoulio General Hospitals, Nea Ionia, 14233 Athens, Greece; virginia@otenet.gr; 14 Department of Systems and Computer Engineering, Carleton University, Ottawa, ON K1S 5B6, Canada; mfrize@gmail.com

**Keywords:** COVID-19 impact, working from home, gender, biomedical fields, qualitative international survey analysis

## Abstract

(1) Background: This paper aims to present and discuss the most significant challenges encountered by STEM professionals associated with remote working during the COVID-19 lockdowns. (2) Methods: We performed a qualitative analysis of 921 responses from professionals from 76 countries to the open-ended question: “What has been most challenging during the lockdown for you, and/or your family?” (3) Findings: Participants reported challenges within the immediate family to include responsibilities for school, childcare, and children’s wellbeing; and the loss of social interactions with family and friends. Participants reported increased domestic duties, blurred lines between home and work, and long workdays. Finding adequate workspace was a problem, and adaptations were necessary, especially when adults shared the same setting for working and childcare. Connectivity issues and concentration difficulties emerged. While some participants reported employers’ expectations did not change, others revealed concerns about efficiency. Mental health issues were expressed as anxiety and depression symptoms, exhaustion and burnout, and no outlets for stress. Fear of becoming infected with COVID-19 and uncertainties about the future also emerged. Pressure points related to gender, relationship status, and ethnicities were also evaluated. Public policies differed substantially across countries, raising concerns about the adherence to unnecessary restrictions, and similarly, restrictions being not tight enough. Beyond challenges, some benefits emerged, such as increased productivity and less time spent getting ready for work and commuting. Confinement resulted in more quality time and stronger relationships with family. (4) Interpretation: Viewpoints on positive and negative aspects of remote working differed by gender. Females were more affected professionally, socially, and personally than males. Mental stress and the feeling of inadequate work efficiency in women were caused by employers’ expectations and lack of flexibility. Working from home turned out to be challenging, primarily due to a lack of preparedness, limited access to a dedicated home-office, and lack of previous experience in multi-layer/multi-scale environments.

## 1. Background

The COVID-19 pandemic has generated an unprecedented challenge of a forced rearrangement of working conditions for a very large number of professionals. Faced with remote working, people had to adapt to the new working conditions involving professional, childcare, schooling, and household duties alike. Social distancing and isolation caused increasing distress that affected both children and adults and was often reflected in their work performance.

While the pandemic has differently affected various professionals regarding remote working, it is assumed that an added difference is related to gender, as the major burden of child, elderly, and other types of care are still predominantly on the shoulders of women.

The implications for gender equity regarding working from home in the United Kingdom during the peak of the first lockdown (mid-2020) were examined by Chung et al. using a survey for working parents [1]. In situations where both partners worked from home, the study concluded that females were mainly responsible for both household and childcare duties in most households, similar to the pre-pandemic times. Furthermore, the increase in housework and childcare for many women negatively impacted their work capacity and efficiency. In households where only males worked remotely, fathers’ involvement in home-schooling and childcare increased compared to pre-pandemic times. The experience of working from home is expected to facilitate a more balanced distribution of unpaid work among couples [1].

According to Feng et al. remote working created a gender gap in perceived work productivity and job satisfaction. Women reported lower work efficiency and fulfilment than men due to multitasking and higher household-related workload [2]. Similar results were reported by Rozman et al. based on a survey conducted on 785 employees in Slovenian companies [3]. Their results showed significant gender differences in work engagement, satisfaction, and efficiency during work from home due to higher household demands, increased stress levels, declined concentration, and the need for multitasking. Investigating the impact of lockdown on dual-earner parents based on a large Australian survey (1536 couples) Craig et al. concluded that while gender gap narrowed to a certain extent due to better implication of fathers in childcare, women were more discontented about work-family balance than before the pandemic [4]. Similar observations were drawn from studies undertaken on other continents [5,6,7].

Contemplating on the possible long-term consequences of home office, Arntz et al. consider that working from home may provide an opportunity for women to catch up with men on work-related aspects; however, in the context of childcare, remote working might accentuate the traditional gender responsibilities [8].

Given the above, the Women in Medical Physics and Biomedical Engineering (WiMPBME) task group of the International Union for Physical and Engineering Sciences in Medicine (IUPESM) has designed and conducted a survey on gender-related work from home under the umbrella of the three main international organisations of our professions: (1) IUPESM, (2) the International Federation of Medical and Biological Engineering (IFMBE), and (3) the International Organization for Medical Physics (IOMP). The quantitative data originating from the survey has been interpreted and published [9].

Based on the 921 responses from biomedical professions, a subset of STEM professionals, representing 76 countries, this paper aims to present and discuss some of the qualitative answers addressed by the survey participants, more specifically the most challenging aspects encountered by professionals, both males and females, during the COVID-19 lockdown regarding remote working.

## 2. Methods

### 2.1. Data Collection

This evaluation focuses on responses to one open-ended question from a larger survey evaluating the impact of the COVID-19 pandemic on gender-related work from home in STEM fields [9]. The question asked and analysed for this study was “What has been most challenging during the lockdown for you, and/or your family?” Participant data was collected from self-selecting volunteers using the SurveyMonkey platform, disseminated worldwide through professional and personal email contacts among members of the WiMPBME task group. It was also promoted through web postings and social media of organisations involved. Specific methodology related to data collection, ethics approvals, and relevant processes has been described elsewhere [9]. The raw data was exported from SurveyMonkey to Microsoft Excel 2016 and subsequently imported into NVivo version 1.0 (2020) for analysis. 

### 2.2. Coding

A codebook was developed by one coder (KVC) who reviewed all raw response data prior to any coding and created a list of thematic units, which was collapsed to form key themes. Key themes and sub-themes were then tailored further in response to data review by a second coder (EB) to produce the final codebook (Appendix A). Thematic units enabled data to be unitised, which is recommended with response data of variable length [10]. This enabled response data from one individual to be categorised under multiple themes. For example, “not being able to leave home or visit my family” was separated into two units, being “not being able to leave home” and “not being able to…visit my family”. Responses to questions were treated as one cohesive dataset with coding and development of analytic patterns performed across all responses, rather than as a response per question. This is in line with recommendations by other researchers when performing qualitative analysis of survey responses [11].

Two independent coders (KVC and EB) coded all the data independently using the pre-specified codebook. They were blinded to participant characteristic details, except where the participant reported these characteristics in their response to the question. Coding comparison through inter-rater reliability testing was substantial according to the Landis and Koch interpretation [12], good according to Altman [13], and very good based on the Fleiss, Levin and Paik [14] interpretation, with a kappa of 0.8013.

### 2.3. Data Analysis

A narrative synthesis of participant responses is provided to summarise key points across each of the identified themes. Descriptive statistics were used to explore coding frequency in subgroup analyses for each thematic unit compared to the pre-specified characteristics of gender, relationship status, and ethnicity using the crosstab function in NVivo. These characteristics were chosen as the most relevant to explore for identification of population differences in participant responses, while minimising risk of type 1 error from purely exploratory comparisons. 

## 3. Results

### 3.1. Characteristics of Participants

From 921 responders to the initial survey [9], 646 responded to the question, “What has been most challenging during the lockdown for you, and/or family?”. As reported in Table 1, these participants included 403 (62%) females, were primarily in the age group of 30–44 years (56%), primarily Caucasian (49.8%), and most participants reported having access to a home office (69.7%). Just under half of the population did not have any children (45.6%), but for those who did the most common number of children was two (27.3%).

### 3.2. Thematic Analysis through Narrative Synthesis

**Figure 1:**
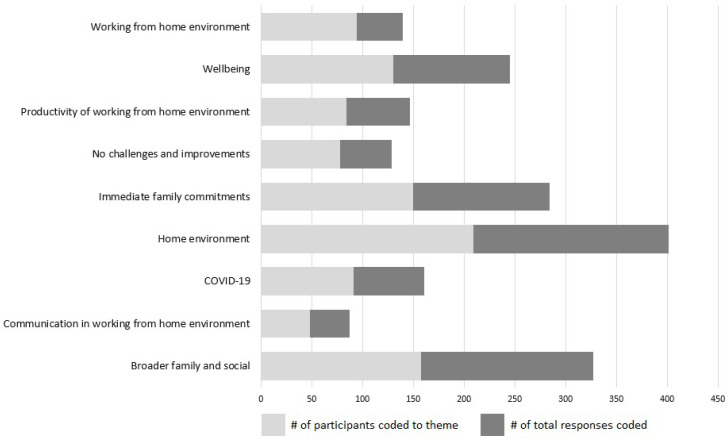
Coding frequency across themes by total participants and number of responses coded.

A total of n = 1921 thematic units were coded using 1042 unique participant references across nine broad themes (Figure 1), producing an average of 1.80 themes coded per participant (with range of 0–6 and mode equal to 1). Of the 646 responders, n = 10 participants were unable to be coded within any theme due to ambiguity or a nonsensical response. The three most reported themes were the immediate family commitments, the broader family and social commitments, and the home/work environment. Through content analysis, a total of nine broad themes and 36 sub-themes were identified, which are described below under their broad themes.

***Immediate family commitments:*** The immediate family unit, being spouses, children and parents who are isolated to the same household, reported a lot of challenges during the lockdown, including care responsibilities, school and childcare responsibilities, as well as concerns over the emotional wellbeing of children. Care responsibilities related primarily to elderly relatives who were unwell and required time and resources by family to support their needs, *“Caring for an elderly relative who couldn’t be assessed for a care package”*.

The school needs of children were a large contributor to lockdown challenges, with concerns about space, time commitments of parents to support schooling, different school requirements across different age groups, lack of support from schools, and guilt that parents weren’t doing enough to support their children’s educational needs, *“Managing online school for 3 children in 3 different schools, with different schedules and expectations”; “…the burden of resolving all problems and answering questions falling to us (instead of teachers)”; “lack of supporting resources to keep the kids having meaningful time”; “Balancing work and home schooling and doing both badly (or at least inefficiently)”; “Guilt that we are doing the bare minimum with our kids and not providing them with a great education”.*

Alongside school requirements for older children, childcare responsibilities needed to be managed in conjunction with large employment workloads. This was a particular concern for people with young children, who would typically rely on external childcare services to free up time for work commitments, *“Trying to keep on top of housework and childcare while also trying to complete required number of work hours”; “Trying to balancing the demands of a very clingy baby, stimulate my pre-schooler, and maintaining my work productivity”; “Caring for a kid is another full time job on top of my actual job”.*

This resulted in unhealthy adjustments to lifestyles, such as extended work hours or sacrifices to work productivity, “*Working evenings and weekends to maximise childcare time”; “Balance of young children and work. I end up working on and off between 7 am and 10 pm depending on the children’s needs”; “Sick days for 1-yr old son. If he is unable to go to day-care, then working from home is a challenge. I try to get uninterrupted time during his nap or after bedtime*”. Parents were also concerned about the impact that these attempts to prioritise work on their children when childcare needed to be balanced in the home, “*Juggling working hours and childcare between me and my husband in such a way that our daughter is not just plonked in front of a TV. I work part time, he works full time. Unfortunately, there are times when we are both working, or in meetings, and she has had to be left to herself. She is only 4, so can’t really expect her to entertain herself for too long*”.

Similarly, the emotional needs of children were cause for concern among parents due to disruption to routines and the removal of social interactions and both psychological and physical outlets supporting wellbeing, *“Managing children’s frustration with the pandemic (especially teenagers)”; “Dealing with mental health issues involving my teenagers”; “Children missing friends”; “The kids are used to having very busy lives, and suddenly, they were left very socially isolated without much to do”.* Parents were then concerned about the alternative avenues of entertainment children engaged in, with screen time activities being the dominant worry, *“Too much screen time for the younger child”; “Getting the youth away from their computers”; “…too many distractions from social media and gaming”.*

Financial worries were also identified by some participants, particularly related to job loss and reduced security in employment, *“Spouse was furloughed then laid off”; “The loss of my husband’s job”; “Not knowing if my job was secure”.*

***Broader family and social:*** The loss of social interactions was identified as one of the biggest challenges faced by the lockdown. People missed visiting family and friends, especially when family members were grown-up children, unwell, or elderly, *“Was usually visiting my old parents and siblings once a month. The most challenging problem was not to even have chance to visit them for more than four months.”* It wasn’t just the social interaction that was missed, but the physical contact of others, *“Lack of human touch. No hugs”; “No kisses to loved ones”; “no physical contact with elderly parents”.*

Some identified the ability to support family members who were struggling with lockdown as a concern, particularly when family members were relying on these visits for their own psychological welfare, *“…especially when family members were grown-up children, were unwell or elderly and relied on these visits for their own mental wellbeing”; “mental strain of concern for family members isolated”.*

People also missed social experiences such as travelling and spending time in social settings and at social events. This was not just organised events, as due to COVID-19 restrictions some public spaces were also closed, *“Not being able to go to church and other activities such as social events, movies etc.”; “Parks, trails and beaches being closed”,* the loss of these social outlets was found to cause behavioural changes from frustrations and reduced external stimulation, particularly among children, *“Cancellation of all social, preschool, and sports (gymnastics) classes and activities for the kids as well as closure of parks and libraries, so the children are confined to the house and yard all day every day. Their behaviour has been harder to manage with the loss of these outlets for activity and socialisation”.*

***Home environment:*** There were many reported changes in the home environment, with people reporting that domestic duties increased, with more housework and the need to cook everyday, due to people being at home more and ordering ready-made food outside of the house being more difficult. For some people to work full-time, they relied on other people to help with domestic duties, which could no longer occur due to lockdown requirements, *“Not having the cleaning lady and having to do home chores”; “I used to have maids and part-timers for daily chores, I now have to do all grocery shopping for a household for 4 because they are all my dependants”.* For most of the responders, the balance of time and demands was the biggest problem, *“Balancing everything. Ensuring the kids are happy. Keeping the house clean. Continuing to run our business”.* People also reported difficulties with usual domestic duties such as shopping and getting essential items, *“There was only a little difficulty in finding free slots for on-line grocery shopping”; “Go shopping food and the protocol to disinfect all”; “Unable to buy essential items”.* These changes to the home environment did cause some negative consequences in the family dynamic, *“The long working hours and time spent on household/pet chores sometimes took a toll on family relationship”.*

Attempts to balance all the usual pre-lockdown activities while in lockdown caused people to feel inadequate as they tried to keep the normal routines in place and struggled to adapt to the new environment, *“Trying not to feel guilty about not always working and instead giving my children some focused attention without trying to multi-task; feeling like my co-workers (without children or with stay-at-home partners) do not understand the struggles of balancing full-time work and childcare; struggling to complete all my work-related tasks in the limited hours I have”*.

In this new home environment, people tried to adjust to constantly being in close quarters with other members of the family all the time and would try to apply boundaries on personal space, *“Seeing too much of each other”; “Family coexistence in respecting individuality”; “Being together ALL THE TIME”; “Small house, all being home in it at the same time with very different hours of awake and sleep time”; “Maintaining private space in a small 3 bedroom house with two adults and four children (the eldest been 20, youngest been 12)”.* This meant that time outside was missed and this was therefore identified as being a substantial challenge, *“To stay in the house for a long time and not be able to go out”; “The stress of not being allowed to do things outside of the house”.* As a result, people reported blurring lines between work and home, which resulted in a feeling of repetition, *“My 400 sq ft living space becoming my workspace as well; there is no work-life separation anymore and feeling of living the same day over and over set in early. Depression and anxiety run high”.*

***Working from home environment:*** *“Work is not work; home is not home”.* Many people found time issues to be the biggest concern when trying to work from home. This included issues of trying to find time to fit everything in, the workday being spread out over longer periods, not knowing when to stop work and resume family time, and blurred lines between what would otherwise be very distinct home and work environments, *“Feeling that the day is too long”; “Working too much. Not knowing when to stop”.* Some participants reported that longer working hours were necessary just to balance all the relevant commitments, *“Working evenings and weekends to maximise childcare time.”; “Don’t have days off. Working from Monday to Sunday, being pregnant, so I can work 60 h a week and at the same time taking care of my daughter since she is not attending to school”; “…my part-time work consumed much more time than normal… There was no way of getting time off from work”.* At the same time, others struggled to find the time needed for work, especially when they have children who need care or help with schooling, *“Hard to have dedicated quiet time. Kids keep calling ‘Mum’”; “To have protected and extended time for myself to do my work. I can only have snippets of time* e.g., *30 min–1 h in between management of the kids, their wellbeing, and zoom classes, to do my own work. Work that require longer focus time, would need to wait until after 11 pm, after the kids slept, for me to embark on a 2–3 h of work. Sometimes sleeping only at 2.30 am. It is very taxing and certainly unsustainable everyday”.*

Finding adequate workspace in the home was also a problem, especially when space is needed for adults working from home and for children doing schoolwork, *“Having the whole family working/schooling from home, so finding suitable space for everybody”; “…not enough computers in the house for everyone to work efficiently all the time”; “Not being disturbed because I had to work in my dining room”.* This issue sometimes caused friction and comparisons about whose work was more important to prioritise for the available space, *“Challenge of negotiating whose work is more important at any given time”; “Finding the balance between my wife’s work (Fed Gov) and my work (Healthcare). The discussion determined that my work in Healthcare was more important and that I be given priority to continue the work for our community, hospitals, testing centers, etc.”* It was also a problem when work required confidential discussions, *“…lack of privacy and space, such as discussing sensitive issues at work over zoom, or dealing with colleague who is melting down over zoom”.*

Others reported that worksite contact was still necessary. However, the need to work from home made it difficult to track who was in the office and decide when to go into the office. There was also a sense of unfairness when people from some institutions could work from home whilst others were forced to stay in the workplace, and vice versa, *“Keeping track of who is at home versus who is in the clinic on any given day”; “Seeing other institutions work from home and you can’t”.*

***Productivity of working from home environment:*** Some participants reported that employer expectations for output did not change despite the new working environment and some employers had concerns about the ability for work to be done from home, *“Expectation to care and teach children while still completing full or nearly full workload. Managers are trying to be accommodating but the work doesn’t go away”; “School closed, my department not allowed me working from home and no annual leave allowed, this make me difficult because no one take care my children”; “My boss had anxiety about work from home. He was very stressed about our availability* etc. *He is very old school in his views on work/life balance”.* The loss of professional interaction also meant that acknowledgement for work was affected, *“Lack of face to face recognition for doing work”.*

For some participants the expectations of employers increased, *“…the expectations were that we had to be flexible for our students, meaning that in effect we had to be available 24/7 to answer emails and help with their study concerns. In practice, this translated to continuous monitoring of email/online forums and a new chat group setup by a colleague”; “pressure from employer to take leave even though workload has not dropped”; “triple workload!*”. There also appeared to be a significant disparity between participants with children and those without children. “*Expectation to care and teach children while still completing full or nearly full workload. Managers are trying to be accommodating but the work doesn’t go away”; “Redeployment in the hospital. Since I don’t have a family to care for, I was asked to continue my normal job while also being redeployed, resulting in working 60–70 h weeks for the past 2 months. I’m exhausted*”.

The ability to concentrate on work activities in the working from home environment was reported by many participants to be a challenge. Distractions from spouses and children were the primary source of lost concentration, *“My husband and I distract each other while working from home. It is harder to focus”; “Being interrupted every hour by our 9 yo* [years old] *needing something”; “I am supposed to help them with virtual learning as school is closed. Keep getting disturbed so cannot focus on my work. My husband also trying to work. We have no home office. When one is talking (e.g., Zoom meeting), it is difficult to other. Hated tele-meeting online”.* While distractions around the home related to usual hobbies, housework, and pets, *“There are definitely more distractions from home that took adjustment (i.e., cats jumping on home desk while working)”.*

Participants identified that adaptations were needed to manage the workload, otherwise productivity decreased, “*Combine the time of work, rest and home duties, because there is a lot of tasks and is essential optimise the organisation of it*”. Unfortunately, in some cases, it was simply not productive to work from home, which had significant negative ramifications for productivity, “*Seminars were cancelled by clients”; “laboratory activities were a challenge for me. I cannot pretend that my students have gained technical skills”; “All the working schedules of onsite training got affected, even though we changed to deliver the training remotely, but we can’t provide the full content to our user effectively and we can’t be sure that they can operate the machine on their own*”. This was a particular issue among teachers who were required to quickly adapt to the new online mode of education delivery with limited success: “*Learning to do online classes”; “Teaching issues”. However, for some, certain working activities had to stop. “No access to lab for work purposes”; “…delayed projects due stopped economy”; “Laboratory work was stopped for some time”; “Not able to access the labs and most experimental works for research are stopped*”.

***Communication in working from home environment:*** Connectivity issues were the biggest barrier to effective communication when working from home. This included issues around no internet connection or slow internet speed, getting access to the virtual private network (VPN i.e., remote access to work computer files) and needing to communicate via phone or e-mail instead of face-to-face, *“Working with those not able to participate in online meetings and thus working with them* via *phone & email”; “Poor internet connectivity to the work environment”; “Accessing files stored on my work computer”.* To address these issues, some people found that the costs of communicating were higher, *“Cost of Internet usage”.* Or they had to develop new ways of communicating, *“…transform the traditional lectures to a lecture assisted by Internet”.*

However, despite attempted workarounds, the loss of professional interaction remained an issue and hindered the ability to effectively communicate, *“To speak to people* via *any video conferencing facilities. The message cannot be delivered effectively”; “Not having in person contact with some colleagues that led to some instances of missed information hand off”; “lag time interaction with peers”.*

One participant felt that changes in communication due to social distancing have had substantial negative ramifications resulting in cultural changes, *“No direct contact with colleagues and students, means that a whole category of spontaneous communication, including questions, incidentals, comments, relationship building, brainstorming, body language, etc., no longer takes place and interactions become stilted, limited and distant. This has successfully engendered fear and distrust. I find this cultural change to be oppressive and difficult to cope with”.*

***Wellbeing:*** Mental health was another area of concern reported by a large proportion of participants. This included worsening symptoms of anxiety and depression, exhaustion and burnout, increased concerns about isolated family members, and not having the usual outlets for stress, *“Mental strain of concern for family members isolated”; “The anxiety and depression. I already had symptoms of anxiety before the quarantine started and I had medical tests scheduled, but I had to cancel everything”.* Participants also identified that a large proportion of concerns related to mental health were due to social isolation, loneliness and cabin fever, *“…the anxiety and stress of confinement”; “Isolation, having to do it all by ourselves”; “Living in the same small place”.* However, for others, the lack of a support network during difficult times and the ability to grieve with family and friends caused the most distress, *“My son was stillborn and we had no family in the state and therefore no physical support for me or my wife from family”.*

For some, it was difficult to mentally switch off because of the blurred lines between the home and work environment, meaning that recovery time was impacted, *“The rest/sleep I did manage to get was interrupted because I couldn’t switch off and get to sleep or would fall asleep only to wake up in the early hours and start thinking about work. There was just no respite, my mind was racing for weeks on end, plus the worry of COVID itself and constant bad news—it was tough”.*

Physical health was also impacted. People were concerned about trying to stay healthy, especially if they already had underlying health issues, “…*diabetic care for ownself*”. However, new physical health issues were identified due to new working from home environments and due to stress, *“I have developed wrist pain from my less ergonomic home office setup”; “…staying at home many times has gave me some sickness like: lack of oxygen in my system and mostly in my cardiac functions... Not going to jogging regularly that produce a lot of stress I am still suffering Now. I am now diagnose with stomach pain since this pandemic started and my doctor diagnose the cause or it coming from stress”.* Linked to physical health were limitations on exercise, which was particularly concerning for people who had routines heavily focused on physical activity, *“Not being able to go to the gym. My entire family spends time at the gym almost daily”; “Lack of space for physical exercises”.*

***COVID-19:*** Many people were concerned about protection from COVID-19, particularly when they themselves or family members were at higher risk of infection due to underlying health concerns, or they were at higher risk of exposure due to their working environment, *“I have an immunocompromised family member. My going into the office on non-remote weeks gives everyone anxiety”; “Managing my own exposure risks during my commute and time spent at work”; “The additional stress of worrying about my immune compromised husband”; “…being sure nor to bring COVID from hospital to home”.*

People were also very mindful of public policies that differed substantially across countries, from no lockdowns in some countries to concerns that civil liberties were being taken away too easily. There were also concerns that they were being expected to adhere to unnecessary restrictions, and similarly, that current restrictions were not tight enough, *“Unreasonable, panic driven decisions and rules. Wrecking the world economy. Watching how easily we are willing to give up freedoms and civil liberties.”;* *“Watching the country fail miserably at handling the pandemic; seeing other countries put more restrictive measures in place (and then see those measures pay off). Knowing that even if we follow the measures put in place in the UK exactly it is still not enough”; “Never being in lockdown, being unable to relate to everyone else in the world because nothing in my work or life changed, no lockdown, no restrictions”.*

The uncertainty of COVID-19 was explicitly mentioned by quite a few participants. This included uncertainty about the future, concerns about getting infected and what this would mean for them and their family, and uncertainty about whether to believe health officials and believing what is said by purported experts through the media, *“Dealing with the uncertainty including while waiting for test results for COVID-19”; “Planning for the uncertainty of the future has been the hardest part”; “…trying to understand the profound changes that the pandemic will bring to our lives”; “Believing what the WHO and others experts say”; “To stay quiet and see so many fake news and bad news on all aspects of life (economic, heath, politics…)”; “Politically motivated news”.*

A few responders reported wearing personal protective equipment such as masks as challenges, while others reported that they or a member of their family had become ill with COVID-19. For example, *“To dress properly with all protective equipment, and wear it all day”; “Partner ill with covid19”; “…my grandfather died for Covid and we couldn’t say goodbye”.*

***No challenges/improvements:*** A substantial number of participants reported no increased challenges and some even identified improvements as a consequence of the lockdown. People without young children or school commitments found productivity increased due to less socialisation and less time spent getting ready for work in the morning and commuting. Some people also identified that confinement to close quarters resulted in building stronger relationships with family and spending more quality time together, *“I’m actually more productive during the lockdown and get to spend more quality time with my household members. I limit my social media and news time to less than an hour a day to avoid becoming anxious about the pandemic. I do not check my e-mail outside of work hours to help establish a boundary between work and home life since I am working from home. I only screen my e-mails for ones from my boss who very rarely e-mails me outside of normal working hours.”; “I have been enjoying working from home and hope to keep it going after the pandemic. It has improved my life allowing me to be more efficient. Less time spent on getting ready [and] traffic”; “I love how much more I’ve gotten to see my kids”; “I thoroughly enjoy working from home, and find myself to be more productive than working in the clinic (fewer distractions and interruptions)”.* Some participants were even able to see the brighter side of enforced social distancing rules, *“It was a rehearsal for my impending retirement.”*

### 3.3. Subgroup Thematic Analysis Based on Key Characteristic Variables

#### 3.3.1. *Gender*

Females were more likely to carry the domestic burden of home chores and caring for children, which meant they were also more prone to having issues trying to balance time and demands. They were more likely to worry about the emotional needs of their children and were more likely to report missing social interactions. Productivity of working from home was reported lower among females than among males, with the ability to concentrate a big concern. The expectations of employers and co-workers were also reported to be higher with a substantial workload. Females were also more concerned about the uncertainty of COVID and ramifications related to this and were more likely to experience mental health issues.

Among males, they were more likely to identify financial concerns as an issue. From a work perspective, they struggled to maintain effective work practices with limitations to worksite contact and the loss of professional interaction. They were also more likely to report the lack of time outside the house in the fresh air as a challenge, and in terms of wellbeing, it was exercise limitations that were the biggest concern. Males were also more likely to report concerns about not getting COVID-19. Otherwise, they reported no challenges, that nothing had changed, and even improvements due to the lockdown, such as being more productive when working from home. 

#### 3.3.2. *Relationship Status*

Relationships were broken up into three categories, these being single, in a partnership, or married.

Among people who were single, the biggest concerns related to care responsibilities for other family members who were unwell. Work activities were more likely to be affected due to cessation or amendments to regular training and services, and the increased costs of communication were a concern. They were substantially more likely to report social isolation as the biggest issue related to mental health. They were frustrated by the different COVID-19 rules for some people still able to work from the office/allowed to work from home and different rules for different countries. Otherwise, they were most likely to report no challenges, that nothing had changed and even some improvements.

For people living in a partnership, the biggest concerns related to care responsibilities for family members requiring visits, missing social interactions with family and friends, and limitations on travel (particularly international), and social events. The working from home environment was also difficult in terms of finding office space and still needing to perform worksite contact on occasion. People in partnerships were also more likely to report mental health as the biggest concern related to wellbeing, and the uncertainty of COVID-19 had negative impacts on planning for social activities and work.

Married couples found the most significant challenges to be associated with the home environment and care for children. The balance of time and demands was a particular concern, as was schooling, childcare and welfare responsibilities for children. In the working from home environment the biggest struggles related to time and productivity, with difficulties concentrating, connecting to necessary services from the office and the loss of professional interaction. Their biggest concern related to COVID-19 was about not getting it and spreading the disease within the family.

#### 3.3.3. *Ethnicity*

Pressure points according to ethnicities were evaluated for populations with nine or more individuals responding to the question of the main barriers.

African American/Black responders were more likely to report barriers related to travel and social limitations, social interactions, and the inability to support family and friends. Cost of communication, such as through internet connectivity, were a concern as part of the working from home environment, whilst not getting COVID-19 and having the correct personal protective equipment available were the concerns specifically related to COVID-19. They also reported a high burden of care responsibilities for external family, and childcare responsibilities were also a barrier during the lockdown.

The biggest challenges experienced by Caucasian populations were found in the home environment and the balance of demands, be that paid work, housework, needs of the family, children’s schooling, and general wellbeing. This was also reported as the biggest issue by South Asian and South East Asian responders. Contributing to this were limitations to office space and finding the time needed for work activities. In addition, the loss of social interactions and the uncertainty caused by COVID-19 were stress points experienced among Caucasians external from the family unit. In contrast, within the family unit, it was their children’s emotional and school needs, missing family and friends, and mental health.

The Hispanic/Latin population were the most likely to struggle with social isolation and report financial concerns such as job loss. They also were most likely to report that they were diagnosed with COVID-19 or had an immediate family member become ill. Middle Eastern responders identified substantial challenges in the home environment, with increased domestic duties, issues getting supplies, childcare responsibilities, and children’s school needs. Employers’ workload expectations were also high, with the inability to undertake worksite contact hindering progress in this area.

Just under half of the East Asian population identified that nothing had changed, as areas such as Taiwan did not experience any lockdowns at the time of this survey. They were the most likely to report care responsibilities for the family that required visits, and their biggest challenges related to the observation that there were different COVID-19 rules across different countries. Meanwhile South Asian responders reported concerns about the uncertainty caused by COVID-19 and worry about becoming ill with COVID-19. The workload was reported to be very high, with concerns about finding time to undertake all the necessary activities, and they were worried about the emotional needs of their children. The combination of these issues may at least in part explain why they were the population most likely to report mental health concerns. Among South East Asian responders, the most substantial concerns related to the home environment and immediate family. Again, finding the time to balance all the demands of work, childcare responsibilities (particularly for young children) and the school needs of older children were reported as the biggest challenges. Productivity of working from home was problematic largely due to issues finding office space; however, problems faced when delivering training and services meant that some had to be altered while others were cancelled altogether.

## 4. Discussion and Conclusions

The results of our international survey clearly showed discrepant outcomes among genders related to home office during the COVID-19 lockdown. Males and females had different standpoints on the positive and negative aspects that originated from remote working conditions, females being overall more affected professionally, socially, and personally than males. Mental stress and the feeling of inadequate work efficiency in women were often caused by their employers’ expectations and lack of flexibility regarding working hours. According to the authors’ previously published results on the quantitative data of this survey, 52.5% females vs. 43.3% males reported that they spent more time working from home as opposed to working from office. Furthermore, while 10.3% males considered that their home office working time has doubled, nearly 18% of females stated that they spent twice as much time working from home as compared to office work [9].

In the circumstances such as the current pandemic, it is not only the employees who have to adapt to new situations and adjust their work accordingly, but it is the duty of the employer as well to provide adequate support to all staff in terms of technical assistance, training (if working from home requires new skills) and wellbeing. An important aspect that employers should consider for both genders in the case of home offices is having flexible working hours (whenever possible) which would ease the burden felt, especially by females when they have to care for small children. While allowing for flexible working hours is more attainable within a company than in healthcare or academia, larger departments could accommodate such schedules with judicious preparations.

Among female academics, burnout during lockdown was often reported due to long working hours, research delay due to lab closure, insecurity, and emotional stress. Expectations from academics to work 24/7 are clearly unreasonable. We conclude that students need to understand that in such unprecedented conditions, they must make an effort to work more independently without relying on teachers’ availability around the clock. For stress mitigation, mental-health consultants have a few recommendations that can bring balance to one’s professional life: (1) find ways to detach from stress by undertaking activities that create a sense of wellbeing; (2) do not internalise burnout as a failure; (3) fight the isolation; and (4) do not focus your conversations on mental health and stress [15].

Working from home turned out to be challenging at the very beginning of the pandemic, primarily due to a lack of preparedness, limited access to a dedicated home-office, and lack of previous experience in such multi-layer/multi-scale environments. However, working from home efficiency grew with time as individuals and institutions built more reliable working procedures. Although the situation was improving from the conjuncture side, the operational side suffers mainly due to the blurring of the borders between professional and personal life and all the sequels this carries. 

Lockdown and social distancing had an immense impact on our daily routines leading to behavioural, cultural and functional changes. While these can be classified as diametrically opposed, their influence over individuals’ well-being in terms of mental and physical health is indisputable.

The global pandemic outlined the weakness of the academic system globally while also turning into a major stimulus for further technological development, improvement of communications, and collaboration [16].

While this pandemic found many universities from around the world unprepared for online teaching, thus disrupting education and research [17,18], it is probably time to learn some lessons and be prepared for any future events that might require similar settings. Digital teaching and learning have evolved dramatically over the past year and should continue to develop so all can embrace the digital system without considering remote schooling a drawback. Universities must keep offering guidance and training to lecturers worldwide to make them accustomed to e-learning tools and feel confident with the online teaching elements. Furthermore, governments must provide reliable and affordable internet connections and access to digital devices to facilitate working from home irrespective of profession, age, and gender.

## 5. Limitations

This study has several limitations. Firstly, as with any survey, there is a response bias where participants with strong opinions are most likely the ones to contribute data to the question. In addition, representation from high socioeconomic countries was more prevalent than from low-socioeconomic countries. Therefore, the generalisability of findings is limited, but they still provide important insight across a broad range of STEM professionals worldwide.

Data analysis for qualitative studies is subjective and was limited by only having two independent coders analyse all the data; however, a high agreement between them was observed on inter-rater reliability testing.

In conclusion, we have presented and discussed the most significant challenges resulting from the COVID-19 pandemic on gender-related work from home in STEM fields- qualitative survey analysis. Our international survey findings indicate that women were more affected professionally in balancing their work, family responsibility, and mental wellbeing than men. Since most of the respondents were obtained from high socioeconomic countries and have shown that the pandemic has a great impact on society, it is highly recommended to further this study and focus on low socioeconomic countries.

We hope that the issues and challenges resulting from COVID-19 presented in this paper will give insight scenarios faced by the employees working from home to employers, government, and institution organisations. For future sustainability, initiatives to introduce and implement a continuous program to overcome these current challenges are highly recommended to have an equal balance of gender-related work from home in STEM fields.

Although the results are based on the challenges faced by health professionals during remote working in the COVID-19 pandemic, the outcome of this survey could be transposed to other unforeseen circumstances when working from home is required. Despite all adversities, for a number of people, the lockdown has offered an opportunity for personal development, discovering new opportunities and new ways of professional growth, considering working from home the bright side of the lockdown.

## Figures and Tables

**Table 1:** Demographic characteristics of survey participants.

	Lockdown Challenge Subset (*n* = 646)		Total Population (*n* = 921)	
	*n*	%	*n*	%
**Gender**				
Female	403	62.4	573	62.2
Male	239	37.0	339	36.8
Prefer not to say	4	0.6	8	0.9
Other	0	0	1	0.1
**Age in years**	*n*	%	*n*	%
18–29	60	9.3	110	11.9
30–44	359	55.6	501	54.4
45–59	167	25.9	231	25.1
60+	53	8.2	66	7.2
Retired	7	1.1	10	1.1
Prefer not to say	0	0	3	0.3
**Racial and ethnic identity**	*n*	%	*n*	%
African American/Black	13	2.0	22	2.4
Caucasian	322	49.8	407	44.2
East Asian	70	10.8	119	12.9
Hispanic/Latin	49	7.6	75	8.1
Middle Eastern	9	1.4	14	1.5
Pacific Islander	1	0.2	1	0.1
South Asian	27	4.2	34	3.7
South East Asian	91	14.1	141	15.3
Prefer not to say	19	2.9	38	4.1
Multiple selections	45	7.0	70	7.6
**Relationship status**	*n*	%	*n*	%
Single	119	18.4	199	21.6
Partnership	81	12.5	117	12.7
Married	439	68.0	587	63.7
Prefer not to say	7	1.1	18	2.0
Home office access *	*n*	%	*n*	%
Yes	450	69.7	543	66.2
No	171	26.5	249	30.4
Partially/limited	25	3.9	28	3.4
**Total number of children** ^†^	*n*	%	*n*	%
0	272	45.6	326	47.0
1	98	16.4	113	16.3
2	163	27.3	184	26.6
3	50	8.4	54	7.8
4	10	1.7	13	1.9
≥5	3	0.5	3	0.4

Key: * *n* = 820 of the *n* = 921 total population participants responded to this question, whilst all *n* = 646 responded to the question for the lockdown challenge subset; ^†^ *n* = 693 of the *n* = 921 total population participants responded to this question, and *n* = 596 of the *n* = 646 lockdown challenge subset responded to this question.

## Data Availability

The data presented in this study are available on request from the corresponding author. The data are not publicly available due to privacy reasons.

## Appendix A. Code book

Immediate family commitments.

Care responsibilities: for sick children, spouses, parents and needs of pets.

School needs of children: Home schooling for children, technology set ups for schooling.

Childcare responsibilities: Children needing childcare at home, to be entertained, needing attention.

Emotional needs of children: children struggling with lockdown, children missing friends/family, concerns over child’s wellbeing, getting children away from technology.

Broader family and social.

Care responsibilities requiring visits: sick parents, grandparents and other family members.

Social interactions: missing family and friends, missing other people.

Supporting family and friends: helping family and friends who are not adjusting to the lockdown.

Limited travel and social events: not able to travel, closure of recreation sites such as beaches and parks, no church, movies or social activities, limited transportation options.

Financial: Job loss, partner laid off, cash/financial concerns.

Home environment.

Balance of time and demands: trying to balance demands of children, work, household needs and other activities, cooking and cleaning merge with work, blurring lines, disruption to routines.

Domestic duties: doing activities at home becomes more difficult, housework, food and shopping, unable to buy domestic items.

Respecting family: trying to co-exist and respect individuality, close proximity to each other all the time, tension at home, personal space.

Time outside: trying to get time in fresh air, not able to go outside.

Comfort: uncomfortable in the home, temperature and trapped inside, small space, doing things for yourself such as reading a book.

Working-from-home environment.

Time: no days off, spread over longer periods, determining whose work is more important to prioritise, drudgery of same routine over time, shift work.

Office space: where to set up an office, not enough computers, getting access to computers, noise, not ergonomic, getting office supplies.

Worksite contact: still needing contact visits for worksite, keeping track of office vs. home working staff, not being able to work from home.

Productivity of working-from-home environment.

Expectations from employers/co-workers: meeting deadlines, no consideration for new environment, moody boss.

Concentration: regular interruptions from family as well as distractions from things needing to be done at home.

Workload: lots to do, multi-tasking, productivity falling.

Cessation or amendment to training/services: some skills and activities could not be done remotely.

Communication in working-from-home environment.

Connectivity issues: video conferencing poor, internet connectivity/speed poor, getting VPN access.

Costs of communicating: internet costs.

Revolutionising communication: transforming traditional lectures to lecture assisted by internet.

Loss of professional interaction: personal interaction loss, missed information.

Wellbeing.

Mental health: stress, anxiety, burnout, fatigue, guilt, loneliness.

Social isolation: same environment over and over, cabin fever, boredom.

Physical health: weight gain, diagnosed health issues, cancelling health appointments.

Exercise limitations: unable to exercise, lack of space for exercise.

COVID-19.

PPE: Wearing masks, wearing PPE, minimising risks.

Uncertainty: about the future, believing what the experts say, not agreeing with public policy decisions, moving countries or workplaces and uncertainties with this.

Different and new COVID rules: Some countries have different rules: some countries do not have lock downs, some people do not abide by COVID rules, changes due to COVID, waiting everywhere, needing identity checks because of COVID.

Not getting COVID: scared, trying to reduce risk of getting infected, understanding what lifestyle changes are needed, high risk of getting COVID, quarantine.

Got COVID or immediate family member got COVID.

No challenges/improvements.

Nothing changed: no changes, no difference.

Improvement occurred: better communication, happier, more time with family, better work practices such as breaks, spending more time with family.
